# Impact of *EFEMP1* on the survival outcome of biliary atresia in Thai infants

**DOI:** 10.1038/s41598-022-19457-1

**Published:** 2022-09-16

**Authors:** Wison Laochareonsuk, Kanita Kayasut, Komwit Surachat, Piyawan Chiengkriwate, Surasak Sangkhathat

**Affiliations:** 1 Department of Surgery and Translational Medicine Research Center, Faculty of Medicine, Prince of Songkla University, Hat Yai, 90110 Songkhla Thailand; 2 Department of Pathology, Faculty of Medicine, Prince of Songkla University, Hat Yai, Thailand; 3 Department of Biomedical Science and Biomedical Engineering, Faculty of Medicine, Prince of Songkla University, Hat Yai, Thailand; 4 Department of Surgery, Faculty of Medicine, Prince of Songkla University, Hat Yai, Thailand

**Keywords:** Molecular biology, Gastroenterology, Molecular medicine

## Abstract

Genome-wide association studies (GWASs) have identified a genetic associated between *EFEMP1* and biliary atresia (BA). To examine the susceptibility of single nucleotide polymorphisms (SNPs) in *EFEMP1* in Thai BA patients, we performed an analysis of the genetic associations and biological interactions with previously reported key SNPs in *ADD3*, a key gene associated with BA. The study also used high-throughput sequencing to detect novel variants in both genes. In addition, the clinical impact of *EFEMP1* SNPs in terms of survival association was also evaluated. The genotypes of 60 BA patients and 179 controls were evaluated using a TaqMan genotyping assay for rs2501577 and rs17095355 in *ADD3* and rs6761893 and rs727878 in *EFEMP1*. The genotype frequencies were analyzed together with the SNP-SNP interactions. Fine mapping by whole-exome sequencing was performed to identify deleterious variants within both genes, and the survival analysis results were analyzed with the *EFEMP1* SNPs. The recessive genotypes of rs2501577, rs17095355 and rs6761893 showed significantly higher frequencies in the BA patients than the controls, and a logistic regression showed that minor alleles of those SNPs increased the BA risk by ORs of 1.86, 1.67, and 1.84, respectively. Moreover, the SNP-SNP interference suggested that a combination of recessive alleles from the 2 genes resulted in an additive risk to BA. In addition, rare missense variants in the gene coding sequences were identified in 7 cases. Immunohistochemical studies revealed a pattern of ADD3 downregulation and EFEMP1 overexpression in the bile ducts of BA patients. Patients with the AA genotype of rs6761893 had significantly lower 5-year native liver survival (34.0%) than those with AT/TT (75.0%), with a log-rank p value of 0.041. Variants in *EFEMP1* are associated with the occurrence of BA in Thai patients. In addition, these variants have an additive influence on BA risk when combined with *ADD3* variants. Moreover, rs6761893 in *EFEMP1* was indicative of survival in Thai BA patients.

## Introduction

Biliary atresia (BA) is a cholestatic disease in infants characterized by progressive fibrosclerosis and inflammatory obliteration of biliary trees^1^. BA occurs in approximately 5 to 14:100,000 livebirths, with the highest incidence in East Asia^2^. Affected individuals typically develop symptoms within the first few weeks of life, and the associated inflammation gradually causes severe obstruction of the bile flow that leads to cirrhosis, liver failure and death within a few years^3^. Hepatic portoenterostomy (Kasai’s operation) is a surgical option providing surgical bypass for the atretic bile duct in BA, although successful drainage can be achieved in only half of the cases^4^. Although the etiology of BA remains unclear, evidence suggests that it could be related to prenatal maternal infection, environmental factors, or genetic susceptibility^2^.

Recently, genetic predisposing factors have become the primary focus for theories on the pathogenesis of BA since a genome-wide association study (GWAS) was introduced as a high-throughput genetic technique. Single-nucleotide polymorphisms (SNPs) within the locus 10q25, a juxtaposition of Adducin3 (ADD3) and X-Prolyl Aminopeptidase 1, were identified to have a significant association with the occurrence of BA in independent populations, including Han Chineses, Thais, and Europeans^5–8^. A recent GWAS conducted in Europeans suggested a second novel candidate SNP on locus 2p16, which is an intronic region of EGF (Epidermal Growth Factor) containing the fibulin extracellular matrix protein 1 (EFEMP1) gene^9^. Biliary trees are composed of numerous cytoskeletal networks that are assembled from spectrin–actin filaments. *ADD3* encodes adducin gamma, which is a subunit of heteromeric proteins in cytoskeletal networks, while the EFEMP1 gene encodes Fibulin-3, which is expressed in the extracellular matrix^10^. Taken together, the functions of these 2 genes might be interrelated when they play roles together during developmental remodeling of the bile ducts. The ADD3 and Fibulin-3 proteins are expressed in normal biliary epithelium and significantly altered in liver tissue from BA patients. Developmental defects in the intrahepatic biliary tree and decreased biliary functions were observed in *ADD3*-knockdown zebrafish^11^.

Associations with BA have been reported for 2 SNPs in *ADD3* (rs2501577, rs17095355) and 2 SNPs in *EFEMP1* (rs6761893 and rs727878). In this study, we aimed to evaluate any associations between variants in these 4 SNPs in the *ADD3* and *EFEMP1* regions and BA. In addition, fine mapping for low prevalence variants in the coding region of both genes was explored by whole exome sequencing (WES). Additionally, SNP-SNP interactions were analyzed for additive biological influences that might support functional relations between those 2 genes. The expression and localization of the ADD3 protein and EFEMP1 protein were also visualized by immunohistochemistry. In addition, a clinical correlation analysis was performed for native liver survival outcome with the genotypes in both genes.

## Results

### Genotyping and genetic association

The rs2501577, rs11194981, rs6761893 and rs727878 SNP genotypes were identified in 60 patients and 179 controls. The female-to-male ratios in the cases and controls were 1.2:1 and 1.4:1, respectively. The clinical features of the cases are displayed in Table 1. The distributions of those SNPs were in Hardy–Weinberg equilibrium. The recessive genotype frequencies in the controls of the SNPs rs2501577(GG), rs1119498(CC), rs6761893(AA), and rs727878(CC) were 0.19, 0.20, 0.02, and 0.11, respectively. When statistically analyzed by Pearson’s correlation, the 2 SNPs in the *ADD3* regions and the SNP rs6761893 within the *EFEMP1* regions were significantly associated with BA (Table 2). A univariate logistic regression using recessive models showed that rs2501577 (AA/AG vs. GG), rs11194981 (CC/CT vs. TT), rs6761893 (TT/TA vs. AA) and rs727878 (TT/TC vs. CC) increased the susceptibility risk of BA at ORs of 3.73 (95% CI 1.98–7.01), 2.56 (95% CI 1.35–4.84), 6.73 (95% CI 1.94–23.24) and 0.64 (95% CI 0.34–1.19), respectively. However, when the analysis was performed using a dominant genotype model, a statistically significant association was not observed between these genotypes and disease susceptibility. The SNP genotyping and allele distribution are shown in Table 2.

**Table 1:** Clinical demographic data and genotype information of biliary atresia patients.

	Jaundice improved (n = 26)	Jaundice not improved (n = 34)	Total (n = 60)	*P* value
**Sex**				
Male	11 (42.3%)	16 (47.1%)	27 (45%)	0.714^a^
Female	15 (57.7%)	18 (52.9%)	33 (55%)	
Age at surgery (mean ± SD)	86 ± 38	80 ± 25	83 ± 32	0.225^b^
**Serum bilirubin before surgery**				
Total bilirubin (mean ± SD)	12.65 ± 3.94	11.62 ± 4.20	12.20 ± 4.05	0.257^b^
Directed bilirubin (mean ± SD)	11.24 ± 3.69	9.88 ± 3.67	10.65 ± 3.71	0.216^b^
**Serum bilirubin after surgery 1 month**				
Total bilirubin (mean ± SD)	4.44 ± 3.51	17.57 ± 8.25	10.13 ± 8.88	< 0.001^b^
Directed bilirubin (mean ± SD)	3.64 ± 2.96	15.41 ± 6.13	8.73 ± 7.45	< 0.001^b^
**Liver function test after surgery 1 month**				
AST (mean ± SD)	154.56 ± 75.26	313.96 ± 158.80	223.63 ± 142.12	< 0.001^b^
ALT (mean ± SD)	144.85 ± 161.32	201.85 ± 132.85	169.55 ± 151.15	0.027^b^
ALP (mean ± SD)	613.62 ± 315.76	680.08 ± 560.22	642.42 ± 435.73	0.823^b^
**Status**				
Alive	17 (65.4%)	15 (44.1%)	32 (53.3%)	0.457^a^
Death	9 (34.6%)	19 (55.9%)	28 (46.7%)	
**Genotype**				
rs2501577 (A/G)				
AA-AG	21 (61.8%)	11 (42.3%)	32 (53.3%)	0.134^a^
GG	13 (38.2%)	15 (57.7%)	28 (46.7%)	
rs11194981 (C/T)				
CC-CT	13 (50.0%)	24 (70.6%)	37 (61.7%)	0.104^a^
TT	13 (50.0%)	10 (29.4%)	23 (38.3%)	
rs6761893 (T/A)				
TT-TA	25 (96.2%)	27 (79.4%)	52 (86.7%)	0.059^a^
AA	1 (3.8%)	7 (20.6%)	8 (13.3%)	
rs727878 (T/C)				
TT-TC	18 (69.2%)	24 (70.6%)	42 (70.0%)	0.909^a^
CC	8 (30.8%)	10 (29.4%)	18 (18.0%)	

*SD* standard deviation, *AST* serum aspartate transaminase, *ALT* serum alanine aminotransferase, *ALP* serum alkaline phosphatase.

^a^Chi-square test or Fisher exact test; and ^b^t test or Wilcoxon signed rank test.

**Table 2:** Statistical analysis of single nucleotide polymorphisms in ADD3 and EFEMP1 among biliary atresia cases and controls.

SNP ID	Gene	Allele	Genotype						P value	Recessive model (rr plus rr′ vs. r′r′)	
			Case N = 60 (%)			Control N = 179 (%)					
			rr	rr′	r′r′	rr	rr′	r′r′		OR (95% CI)	P value
rs2501577	*ADD3*	A/G	12 (20.0)	20 (33.3)	28 (46.6)	55 (30.73)	90 (50.28)	34 (18.99)	< 0.001	3.73 (1.98–7.01)	< 0.001
rs11194981	*ADD3*	T/C	14 (23.33)	23 (38.33)	23 (38.33)	54 (30.17)	90 (50.28)	35 (19.55)	0.013	2.56 (1.35–4.84)	0.004
rs6761893	*EFEMP1*	T/A	35 (58.33)	17 (28.33)	8 (13.33)	116 (64.8)	59 (32.96)	4 (2.23)	0.003	6.73 (1.94–23.24)	0.002
rs727878	*EFEMP1*	C/T	11 (18.33)	31 (51.67)	18 (30.00)	19 (10.61)	88 (49.16)	72 (40.22)	0.179	0.64 (0.34–1.19)	0.123

The allelotype analysis identified significant associations between BA risk and rs2501577 (G), rs11194981 (C), and rs6761893 (A) (OR 1.86, 95% CI 1.24–2.84, OR 1.67, 95% CI 1.11–2.53, OR 1.84, 95% CI 1.16–2.89, respectively), while the BA risk with SNP rs727878 (T) had an OR of 0.67 (95% CI 0.44–1.0). The pairwise linkage disequilibrium (LD) of all BA-associated SNPs is shown in Supplementary Table 1.

### SNP–SNP interaction

The interaction between SNPs from both genes was analyzed by combining the genotypes from the SNPs with the highest OR from each gene (rs2501577 from *ADD3* and rs6761893 from *EFEMP1*). Cases with minor alleles from both SNPs (A–T) were identified to have a susceptibility risk for BA, with an OR of 9.47 (95% CI 4.22–21.24) in the multivariate logistic regression analysis. In addition, the positive biological interactions of both minor alleles were at RERI 0.54 and AP 0.73, as shown in Table 3. Because combined homozygous recessive genotypes from the 2 SNPs (AA–TT) were not observed among the study population, combined genotypes with minor alleles from the 2 SNPs were combinations of homozygous recessive genotypes and heterozygous genotypes (AG–AA and GG–TA). On the multivariate logistic regression analysis, the combinations showed significantly increased susceptibility to BA at ORs of 7.65 (95% CI 1.79–32.60) and 7.87 (95% CI 2.07–29.95). The biological interaction analysis revealed that *ADD3* and *EFEMP1* had positive interactions with RERI 0.74 and AP 3.03, as shown in Table 4.

**Table 3:** Analysis of the biological interactions between allelotypes in the biliary atresia cases and controls.

*ADD3*	*EFEMP1*	Frequency	Control	Odd ratio	P value
rs2501577	rs11194981	Case			
A	T	31 (18.79)	134 (81.21)	Reference	
	A	19 (24.05)	60 (75.95	1.61 (0.98–2.65)	0.062
G	T	54 (27.14)	145 (72.86)	1.37 (0.72–2.61)	0.342
	A	22 (62.86)	13 (37.14)	7.32 (3.32–16.10)	< 0.001
			RERI	5.34 (− 0.03 to 10.70)	0.051
			AP	0.73 (0.50–0.97)	< 0.0001
			SI	6.45 (1.59–26.15)	0.009

**Table 4:** Analysis of the biological interactions between *ADD3* and *EFEMP1* genotypes comparing the biliary atresia cases and controls.

*ADD3*	*EFEMP1*	Frequency	Control	Odds ratio	P value
rs2501577	rs11194981	Case			
AA	TT	8 (13.33)	35 (19.55)	Reference	
	TA	3 (5.00)	20 (11.17)	0.65 (0.16–2.76)	0.565
	AA	1 (1.67)	0	NA	
AG	TT	8 (13.33)	52 (29.05)	0.67 (0.23–1.96)	0.468
	TA	5 (8.33)	34(18.99)	0.64 (0.19–2.16)	0.476
	AA	7 (11.67)	4 (2.23)	7.65 (1.79–32.60)	0.006
GG	TT	19 (31.67)	29 (16.20)	2.86 (1.09–7.49)	0.032
	TA	9 (15.00)	5 (2.79)	7.87 (2.07–29.95)	0.002
	AA	0	0	NA	
			RERI	0.74 (0.30–1.17)	< 0.001
			AP	3.03 (0.18–5.88)	0.037
			SI	NA	

### Immunohistochemistry of fibulin-3 and ADD3 protein in BA liver

**Figure 1:**
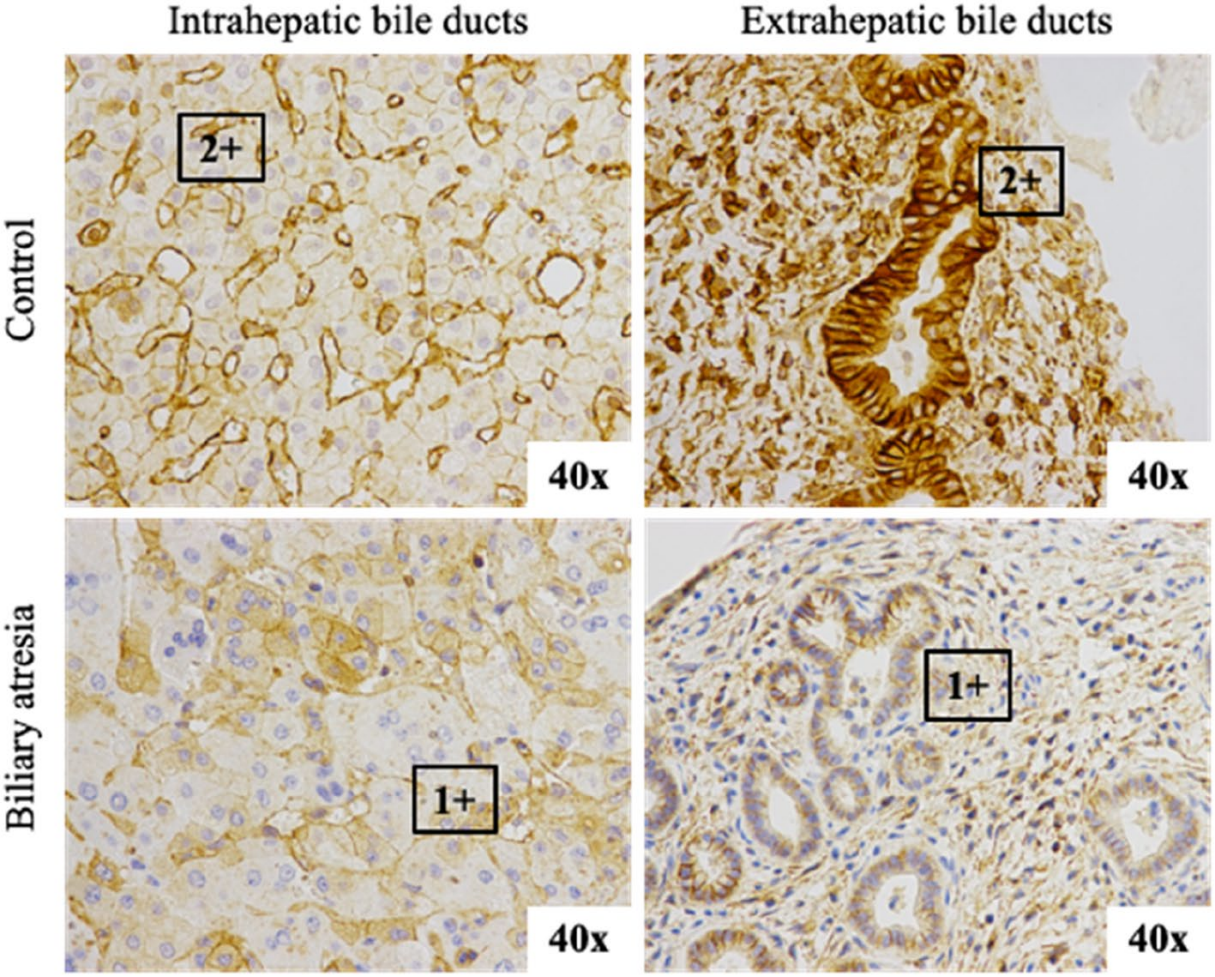
Immunohistochemistry staining of ADD3 proteins in the biliary epithelium showing decreased expression in both areas of the biliary atresia cases.

**Figure 2:**
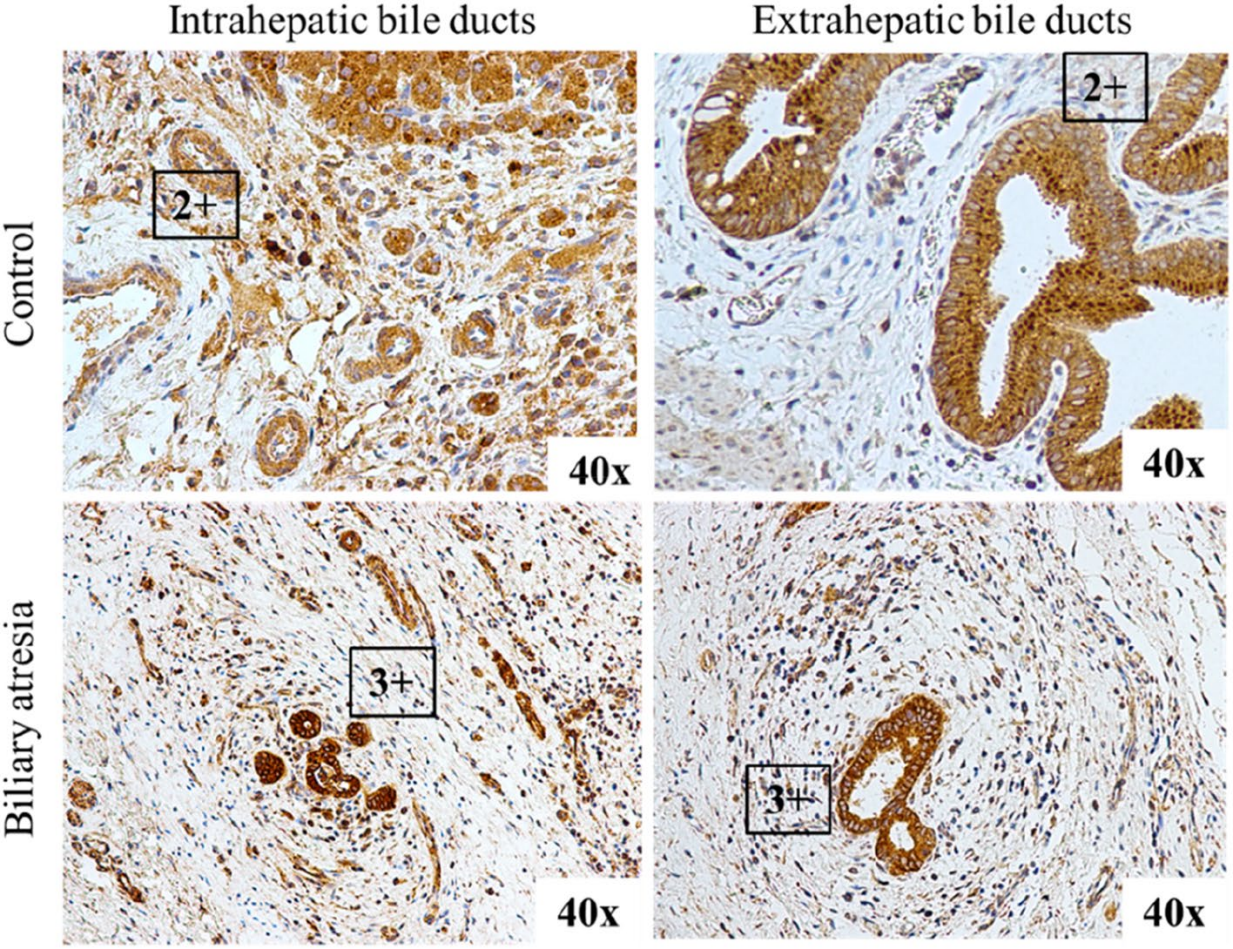
Fibulin-3 immunostaining in biliary epithelium showing the overexpression and loss of apical granules within the intrahepatic bile ducts in biliary atresia patients.

A immunohistochemistry analysis showed that ADD3 and Fibulin-3 are widely expressed in the biliary epithelium, including the intrahepatic ducts and extrahepatic ducts of non-BA liver, as previously suggested. ADD3 staining was localized at the cell membranes and the cytoplasm of the biliary epithelium. In addition, dense granules of Fibulin-3 staining were identified in an apical area of the epithelium. In BA cases, our study suggests that the cellular expression of ADD3 in the hepatobiliary tract decreased or was evenly unexpressed in all cases, as shown in Fig. 1. Interestingly, homogenous overexpression of Fibulin-3 within the intrahepatic duct epithelia was observed in 5 BA cases, in which the intracellular dense granules disappeared in the representative section, as shown in Fig. 2.

### Fine mapping of *ADD3* and *EFEMP1* in BA patients by WES

Fine mapping of variants in *ADD3 and EFEMP1* was performed by WES in 60 BA patients. Rare nonsynonymous variants of the studied genes were identified in 7 cases, as shown in Table 5. A variant detected in *ADD3* (rs371961813) was predicted to cause damage to the protein by all variant predictor tools. Moreover, a missense mutation in *EFEMP1* (rs146446706) was detected in 4 cases and evaluated as possibly deleterious based on Mutation Taster^12^ and CADD^13^ scores. All identified variants were confirmed by Sanger sequencing. Consistent with the clinical characteristics, those patients with identified mutations had poor surgical outcomes in terms of their native liver, and they frequently developed postoperative liver cirrhosis and ending with liver failure. In addition, most of these patients died within the first year of life, as shown in Supplement table S2.

**Table 5:** Rare variants in the ADD3 and EFEMP1 genes identified by whole exome studies.

Case	Chr: position	SNP ID	Gene	Variant Effect	Nucleotide change** (**Zygosity**)**	Amino acid change	MAF	SIFT	PolyPhen2	Mutation Taster	CADD
B001	chr2: 55876743		*EFEMP1*	Splice acceptor variant	c.761-1G>C (HET)						
B011	chr2: 55876743	rs146446706	*EFEMP1*	Missense variant	c.1160G>A (HET)	p.Arg387Gln	0.013	0.27 (T)	0.02 (B)	43, 0.99 (D)	22.6
B029	chr10: 110112826	rs371961813	*ADD3*	Missense variant	c.245G>A (HET)	p.Gly82Asp	0.001	0 (D)	0.998 (P)	94, 1, (D)	28.1
B031	chr10: 110,133,366	rs574688215	*ADD3*	Missense variant	c.1773A > C (HET)	p.Glu591Asp	0.004	0.33 (T)	0.02 (B)	45, 0.99, (P)	28.2
B031	chr10: 110133367	rs541765111	*ADD3*	Missense variant	c.1774G>T (HET)	p.Val592Leu	0.004	0.33 (T)	0.02 (B)	32, 1, (P)	15.94
B056	chr2:55870880	rs146446706	*EFEMP1*	Missense variant	c.1160G>A (HET)	p.Arg387Gln	0.013	0.27 (T)	0.02 (B)	43, 0.99 (D)	22.6
B086	chr2: 55870880	rs146446706	*EFEMP1*	Missense variant	c.1160G > A (HET)	p.Arg387Gln	0.013	0.27 (T)	0.02 (B)	43, 0.99 (D)	22.6
B095	chr2: 55870880	rs146446706	*EFEMP1*	Missense variant	c.1160G>A (HET)	p.Arg387Gln	0.013	0.27 (T)	0.02 (B)	43, 0.99 (D)	22.6

*Chr* chromosome, *SNP* single nucleotide polymorphism, *MAF* minor allele frequency in East Asian populations, *SIFT* scale-invariant feature transform (*T* tolerated, *D* damaging), *PolyPhen* Polymorphism Phenotyping data collection (*B* benign, *P* possibly deleterious, *D* deleterious), Mutation Taster, Mutation Taster–AA change score and probability value (*D* disease causing, *P* polymorphism), *CADD* Combined Annotation Dependent Depletion recorded in Phred score, *HET* heterozygous, *HOM* homozygous.

### Survival analysis

**Figure 3:**
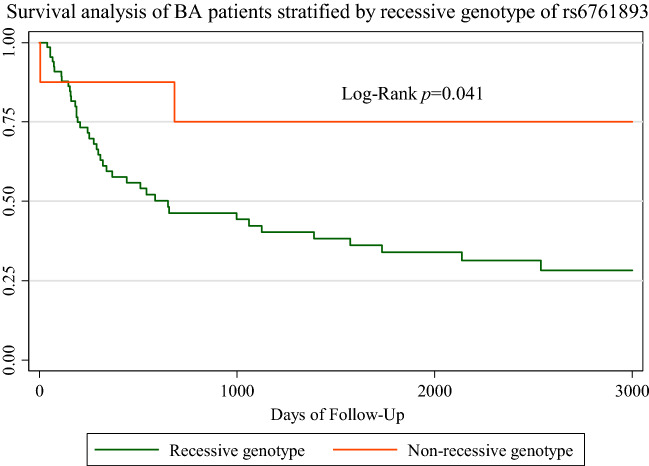
Kaplan–Meier survival plot of the recessive genotype of rs6761893 located in *EFEMP1*.

The survival analysis showed that the 5-year and 10-year native liver survival in all patients was 56.1% and 45.3%, respectively. Survival in those who underwent Kasai’s operation before 60 days of life was significantly better than that in those who underwent surgery later than this cutoff age (p value 0.03) (Supplement Fig. 1). Patients with the AA genotype of rs6761893 had significantly lower 5-year native liver survival (34.0%) than those with AT/TT (75.0%), and the log-rank p value was 0.041 (Fig. 3). On multivariate analysis, the genotype of rs6761893 independently increased hazard risk of survival outcome of the BA (Supplement Table 7).

## Discussion

Biliary atresia is the most common surgically correctable neonatal cholestasis^2^. Severe fibrosclerosis of the bile ducts leads to the blockage of bile clearance, which is potentially treatable by surgical bypass of the extrahepatic biliary system. However, not all BA cases achieve adequate biliary drainage after an HPE operation, and nearly half of BA infants gradually develop progressive periportal inflammation, biliary cirrhosis, and liver failure, which necessitates transplantation^14^. Although the pathogenesis of BA has not been clarified, high-throughput molecular technology potentially overcomes genetic associations in various populations. Previous studies in Thais and other ethnic groups have reported associations between *ADD3* and the disease at ORs ranging from 1.18 to 2.38^5,6,15^. Only one study examining *EFEMP1* SNP associations was available, and it reported an OR of 1.54^9^.

Our study explored genetic associations of *ADD3* and *EFEMP1* and BA in Thai infants using 3 approaches, genetic association studies based on TaqMan genotyping assays, additive biological interactions, and high-throughput genome sequencing to identify novel rare variants in candidate genes. Four SNP genotypes (rs2501577, rs17095355, rs6761893 and rs727878) were examined by TaqMan genotyping assays in both BA cases and healthy volunteers. The genotype frequency of the control population was comparable to that of the HapMap data^16^ and a previous study^8^. Considering the gene pool balance, Hardy–Weinberg equilibrium was confirmed by statistical tests for both the cases and controls. When the genotype data passed the HWE evaluation, the individual genotypes of the SNPs in *ADD3* and *EFEMP1* were analyzed, and significant disease associations were found in homozygous recessive genotypes and recessive alleles. Combinations of the heterozygous recessive alleles were analyzed using an additive interaction model that identified positive interactions between the SNPs from the 2 genes. The identification of positive biological interactions between *ADD3* and *EFEMP1* suggested that the 2 genes potentially shared a role in the pathogenesis of BA.

*EFEMP1* encodes Fibulin-3, which is a component of various extracellular matrix molecules, especially those involved particularly in cell–cell interactions, remodeling, tissue regeneration and embryonic organogenesis. ADD3, a heteromeric protein, is widely expressed in the biliary epithelium and involved in the cytoskeleton network and basement membrane^15^. The protein has functions in cellular adhesion, remodeling, and organogenesis during the embryonic period. In an animal model, *ADD3*-knockout zebrafish developed intrahepatic ductopenia, which led to poor functioning of the biliary system^11^. A previous study identified ubiquitous expression of Fibulin-3 in intrahepatic cholangiocytes^9^. An IHC expression analysis showed that ADD3 expression in both the intra- and extrahepatic biliary tract was decreased or lacking in all our BA cases, and this finding is consistent with a previous study. In contrast, overexpressed EFEMP1 was observed in certain BA cases that had more severe biliary cirrhosis on the histopathology sections.

Considering deep genetic discoveries, fine mapping of coding variants within the 2 genes was carried out by WES. Our study identified not only deleterious and rare variants in *ADD3* but also a rare variant in *EFEMP1*, namely, rs146446706, which was found in 4/60 cases. These variants have never been found in the exome variant database in our population. In addition, all cases with identified variants had poor surgical outcomes and ended with liver cirrhosis and liver failure. The results of the survival analysis, which showed better native liver survival in BA with the recessive genotype of rs6761893 suggested that *EFEMP1* variants might have some defects in biliary tract remodeling during embryonic period^17^ or extracellular matrix dysregulation which may results in decreased bile excretion^18^. Although clear explanation about *EFEMP1* functions is beyond the scope of our study, these findings indicate that *EFEMP1* is an interesting candidate gene that should be further explored for its role in the pathogenesis and severity factor of BA.

In summary, our study validated genetic associations between BA and certain *ADD3* and *EFEMP1* SNPs in the Thai population, confirmed the genetic associations of both genes and demonstrated the interactions using a statistical model. The study detected common mutations of *EFEMP1* in our BA cases and found that patients who harbored the AA genotype in an *EFEMP1* SNP had better prognosis.

## Materials and methods

### Study population

A total of 60 infants with BA were included in our study. The informed consent was obtained from all subjects and/or their legal guardian(s) under permission of the Human Research Ethics Committee of the Faculty of Medicine, Prince of Songkla University (REC-EC-61-268-10-1). All methods were performed in accordance with the relevant guidelines of the ethical committee. Liver biopsy specimens acquired during hepatic portoenterostomies (HPEs, Kasai's operation) in Songklanagarind Hospital between 2003 and 2019 and stored in the Biological Banking System of the Translational Medicine Research Center, Prince of Songkla University^19^ were used for nucleic acid extraction. Informed consent for the use of the tissue specimens was provided by a parent and/or legal guardian. The diagnosis of BA was confirmed by intraoperative findings of obliterated biliary tracts (with or without intraoperative cholangiography) and histopathology of intrahepatic cholestasis. Controls were preserved DNA from 179 sex-matched healthy volunteers residing in the Songkhla area with an age > 15 years and without a history of jaundice or liver disease.

Following HPE, patients were maintained with oral antibiotics for 1 month and ursodeoxycholic acid over a long-term period^20,21^. Oral steroids were used within 2 weeks after the operation. Clinical follow-up and liver function studies were scheduled every 6 months for 5 years and then annually thereafter. Ultrasonography was performed once a year, and esophagoscopy was considered in cases with clinical signs of portal hypertension. Patients with deterioration of liver function were enrolled in the liver transplantation program of Siriraj Hospital, Bangkok.

### Candidate SNP selection

The candidate SNPs rs2501577, rs17095355, rs6761893 and rs727878 were selected from previously published GWASs^5,6,9^. The SNPs rs2501577 and rs17095355 are in the vicinity of *ADD3,* while rs6761893 and rs727878 are in the intronic region of *EFEMP1.* Based on the HapMap database of the Han Chinese population, which is genetically closest to the Thai population, each selected SNP was in a different linkage disequilibrium (LD) block.

### Genomic DNA extraction

Genomic DNA (gDNA) was extracted from the liver biopsy samples using a Qiagen DNA Mini kit (Qiagen, Hilden, Germany). The gDNA of the healthy controls was extracted from peripheral blood leukocytes and banked in a refrigerated environment until use. The specimens were quantified and qualified by a NanoDrop 2000 spectrophotometer (Thermo Scientific, Delaware, United States).

### SNP genotyping assays

Genotyping of the 4 studied SNPs was performed using TaqMan SNP Genotyping Assays run on a 7500 Fast Real-Time PCR system (Applied Biosystems, Foster City, CA, USA). PCR assays (20 µL) was performed in 96-well plates following the manufacturer’s standard protocol. Samples with known genotypes and mock templates were included for each run. The genotype call rate and concordance were assessed using the following criteria: (1) a genotype call rate for each run and overall study > 95%; (2) inclusion of > 10% of duplicates; and (3) concordance rate for the duplicated genotyping > 99%. If any of these criteria were not successfully met, then the experiment was repeated. Representative samples (10%) from each genotype group were submitted for validation by direct nucleotide sequencing.

### Whole-exome sequencing and bioinformatic analysis

Fine mapping of the coding sequences of *ADD3* and *EFEMP1* was carried out using whole-exome sequencing. Exonic regions were captured and enriched using Agilent SureSelect XT Human All Exon v6 (Agilent Technologies, Santa Clara, California, United States). Paired-end sequencing with a 150-bp platform was carried out on an Illumina NovaSeq-6000 (Illumina, San Diego, California, United States) at an average targeted coverage of 100 × depth. Raw reads were aligned with the latest version of the human reference genome sequence (GRCh38.p13) using a Burrows–Wheeler Aligner (BWA-MEM). Variants were identified using a GATK (Genome Analysis Toolkit) HaplotypeCaller, and artifacts were filtered out. Possible pathogenic variants were prioritized using the following criteria: allele depth more than 25% of total reads, reading depth more than 40x, and minor allele frequencies less than 0.01 in East Asian populations. The annotated variants were validated through polymerase chain reaction and dideoxynucleotide sequencing (Sanger sequencing). To predict possible deleterious consequences of identified variants, scale-invariant feature transform (SIFT), Polymorphism Phenotyping data collection 2 (PolyPhen2), Mutation Taster–AA change score and probability value (Mutation Taster) and Combined Annotation Dependent Depletion (CADD) were applied to calculate the damage probabilities, which were interpreted as benign or damaging (SIFT), possibly deleterious or deleterious (PolyPhen2), and disease causing variants (Mutation Taster) and variants with a CADD Phred score over 20.

### Immunohistochemistry

To evaluate the expression and localization of ADD3 and EFEMP1 proteins in the liver tissue and extrahepatic bile ducts, immunohistochemical studies were performed in tissue specimens obtained from the patients’ hepatoportoenterostomy operations. Formalin-fixed, paraffin-embedded specimens of 31 BA cases were provided by the Department of Pathology, Faculty of Medicine, Prince of Songkla University. The controls were liver specimens from nontumoral parts of age-matched hepatoblastoma patients who had no history of liver disease. The 5 μm paraffin sections were sliced onto charged glass slides. The tissue was deparaffinized following a standard protocol. Immunohistochemistry studies for adducin-gamma rabbit polyclonal antibody (Santa Cruz Biotechnology, Santa Cruz, CA) at a dilution of 1:200 and EFEMP1 rabbit polyclonal antibody (Santa Cruz Biotechnology, Santa Cruz, CA) at a dilution of 1:50 were performed on a Leica Bond-III Autostainer (Leica Microsystems, Buffalo Grove, IL). The expression and distribution of the ADD3 protein and EFEMP1 protein were interpreted by a pathologist (K.K.). Expression grading was performed to interpret the protein expression levels, which ranged from 0 to 3+. A level of 2 + indicated equal expression to normal tissue, a level of 0 indicated no protein expression, a level of 1 + indicated decreased protein expression, and a level of 3 + indicated protein overexpression in representative tissues. In general, the structures of bile ducts were identified in Hematoxylin and Eosin stain in most of the cases. The cytokeratin19 (CK19) was stained to demonstrate biliary epithelium in selected cases whose bile ducts were equivocal (Supplement Fig. 2).

### Statistical analysis

The allele frequency of each SNP was calculated using Hardy–Weinberg equilibrium. Associations between SNPs and the occurrence of disease were calculated using the chi-square test, and statistical significance was considered at a p value < 0.05. The risk genotypes were assessed by univariate logistic regression and demonstrated in odds ratios (ORs) with 95% confidence intervals (CIs). Interactions between paired SNPs in the *ADD3* and *EFEMP1* regions were analyzed by combinations of genotypes using a multivariate logistic regression model. The same pairing approach was performed at the allelotype level. Alleles were indicated in binary numbers (0 or 1). The biological relevance of *ADD3 and EFEMP1* on the risk of BA was determined by the relative excess risk interaction (RERI), attributable proportion (AP), and synergy index (SI). A SNP-SNP interaction was considered when RERI > 0, AP > 0, or S > 1. The survival analysis used the log-rank test, and the results are displayed in Kaplan–Meier curves.

## Supplementary Information

Supplementary Information.
