# Supplementary material for: Impact of EFEMP1 on the survival outcome of biliary atresia in Thai infants
Source: Sci Rep. 2022 Sep 16;12:15603. doi: 10.1038/s41598-022-19457-1 (PMC9481615; doi:10.1038/s41598-022-19457-1)
Supplement: Supplementary file 1 — Supplementary Information. [file 41598_2022_19457_MOESM1_ESM.docx]

**Supplement Table 1** Clinical characteristics of affected individuals with genotype TT/TA and AA of SNP rs6761893

|  | **rs6761893 (T/A) genotypes** | | ***P-value*** |
| --- | --- | --- | --- |
|  | **TT and TA  (n=52)** | **AA  (n=8)** |  |
| **Age at surgery** (Mean ± S.D.) | 87.98 ± 4.63 | 73.36 ± 8.04 | 0.291^(b)^ |
| **Serum bilirubin before surgery** | | |  |
| Total Bilirubin   (Mean ± S.D.) | 11.90 ± 3.94 | 11.79 ± 3.07 | 0.938^(b)^ |
| Directed Bilirubin  (Mean ± S.D.) | 10.37 ± 3.64 | 10.50 ± 2.90 | 0.923^(b)^ |
| **Serum bilirubin after surgery 1 month** | | |  |
| Total Bilirubin  (Mean ± S.D.) | 3.59 ± 3.51 | 10.52 ± 8.81 | 0.032^(b)^ |
| Directed Bilirubin  (Mean ± S.D.) | 2.91 ± 3.18 | 9.09 ± 7.43 | 0.023^(b)^ |
| **Liver function test after surgery 1 month** | | |  |
| AST (Mean ± S.D.) | 179.75 ± 105.28 | 228.82 ± 154.51 | 0.386^(b)^ |
| ALT (Mean ± S.D.) | 156.74 ± 106.54 | 245.5 ± 310.76 | 0.094^(b)^ |
| ALP (Mean ± S.D.) | 515.88 ± 425.68 | 646.18 ± 425.68 | 0.407^(b)^ |
| **Status** |  |  |  |
| Alive | 26 (50.0%) | 5 (62.5%) |  |
| Death | 26 (50.0%) | 3 (37.5%) | 0.198^(a)^ |

**Abbreviations:** S.D., standard deviation; AST, serum aspartate transaminase; ALT, serum alanine aminotransferase;
ALP, serum alkaline phosphatase
(a)Chi-square test or Fisher exact test; (b)T-test or Wilcoxon signed rank test

**Supplement Table 2** Linkage disequilibrium of representative single nucleotide polymorphisms

| **SNP-SNP** | | **D'** | | | |
| --- | --- | --- | --- | --- | --- |
|  |  | **rs2501577** | **rs11194981** | **rs6761893** | **rs727878** |
| **R^2^** | **rs2501577** |  | 0.8353 | 0.4089 | 0.1422 |
|  | **rs11194981** | 0.6701 |  | 0.4016 | 0.1936 |
|  | **rs6761893** | 0.0099 | 0.0094 |  | 1 |
|  | **rs727878** | 0.0033 | 0.006 | 0.362 |  |

Supplement Table 3 Expression of ADD3 and Fibulin3 proteins in intrahepatic bile ducts and porta hepatis

| **Case** | **ADD3** | | **Fibulin3** | |
| --- | --- | --- | --- | --- |
|  | **Intrahepatic bile ducts** | **Porta hepatis** | **Intrahepatic bile ducts** | **Porta hepatis** |
| B40 | 1+ | 1+ | 2+ | 2+ |
| B44 | 1+ | - | 2+ | - |
| B45 | 1+ | 1+ | 2+ | 2+ |
| B47 | 1+ | 1+ | 2+ | 2+ |
| B48 | 1+ | 1+ | 2+ | 2+ |
| B50 | 1+ | 1+ | 3+ | 3+ |
| B51 | - | 2+ | - | 2+ |
| B52 | - | 2+ | - | 2+ |
| B53 | 1+ | 1+ | 2+ | 2+ |
| B54 | 1+ | - | 3+ | - |
| B56 | 1+ | 1+ | 2+ | 2+ |
| B64 | 1+ | 2+ | 2+ | 2+ |
| B67 | - | 1+ | - | 2+ |
| B68 | 1+ | - | 2+ | - |
| B70 | 1+ | 2+ | 2+ | 2+ |
| B72 | 1+ | 1+ | 2+ | 2+ |
| B73 | 1+ | 1+ | 2+ | 2+ |
| B79 | 1+ | 1+ | 2+ | 2+ |
| B80 | 1+ | - | 2+ | - |
| B82 | 1+ | 1+ | 3+ | 3+ |
| B83 | 1+ | 2+ | 2+ | 2+ |
| B85 | 1+ | - | 2+ | - |
| B86 | 1+ | - | 3+ | - |
| B87 | 1+ | 1+ | 2+ | 2+ |
| B89 | 1+ | 2+ | 2+ | 2+ |
| B92 | 1+ | 1+ | 2+ | 2+ |
| B94 | 1+ | 1+ | 2+ | 2+ |
| B95 | 1+ | 1+ | 2+ | 2+ |
| B97 | 1+ | 1+ | 2+ | 2+ |
| B101 | 1+ | 2+ | 3+ | 3+ |
| B102 | 1+ | 1+ | 2+ | 2+ |

**Supplement Table 4** Clinical features of BA patients with associated possibly pathogenic variants

| **Case** | **Identified genes** | **Sex** | **Initial bilirubin (DB/TB) (mg/dL)** | **Age at surgery (days)** | **Jaundice clearance** | **Last follow-up age  (status)** | **Last follow-up Initial bilirubin (DB/TB) (mg/dL)** | **Follow-up remarks** |
| --- | --- | --- | --- | --- | --- | --- | --- | --- |
| B001 | *EFEMP1* | F | 12.25/14.60 | 41 | Not improved | 6 months (Death) | 16.23/18.38 | Liver cirrhosis and liver failure |
| B011 | *EFEMP1* | M | 7.65/9.03 | 55 | Not improved | 8 months (Death) | 22.31/26.40 | Liver cirrhosis, liver failure, esophageal varices with recent bleeding |
| B029 | *ADD3* | M | 10.86/11.36 | 73 | Not clear | 13 years (Alive) | 5.08/5.56 | Liver cirrhosis with portal hypertension and esophageal varices |
| B031 | *ADD3* | F | 19.92/19.93 | 38 | Not clear | 7 months (Death) | 7.15/9.79 | Liver cirrhosis with cholangitis |
| B056 | *EFEMP1* | F | 8.84/9.98 | 71 | Improved | 8 Years  (Loss to follow-up) | 1.49/2.05 | Liver cirrhosis with portal hypertension |
| B086 | *EFEMP1* | M | 6.21/7.95 | 54 | Not clear | 10 months (Death) | 3.36/3.76 | Liver cirrhosis and liver failure |
| B095 | *EFEMP1* | F | 16.29/16.80 | 123 | Not clear | 1 years (Death) | 14.50/17.08 | Liver cirrhosis and liver failure |

**Abbreviations:** F, female; M, male; DB, direct bilirubin; TB, total bilirubin

**Supplement Table 5** Subgroup analysis of clinical and genotype data of BA patient classified by age of surgery

|  | **Age at surgery** | | ***P-value*** |
| --- | --- | --- | --- |
|  | **Before 60 days (n=19)** | **After 60 days  (n=41)** |  |
| **Age at surgery** (Mean ± S.D.) | 49.32 ± 9.35 | 99.02 ± 33.95 | <0.001^(b)^ |
| **Serum bilirubin before surgery** | | |  |
| Total Bilirubin   (Mean ± S.D.) | 11.53 ± 4.20 | 12.02 ± 3.84 | 0.618^(b)^ |
| Directed Bilirubin  (Mean ± S.D.) | 9.85 ± 4.20 | 10.57 ± 3.33 | 0.449^(b)^ |
| **Serum bilirubin after surgery 1 month** | | |  |
| Total Bilirubin  (Mean ± S.D.) | 10.97 ± 9.27 | 9.37 ± 8.48 | 0.492^(b)^ |
| Directed Bilirubin  (Mean ± S.D.) | 9.47 ± 8.01 | 8.09 ± 7.15 | 0.483^(b)^ |
| **Liver function test after surgery 1 month** | | |  |
| AST (Mean ± S.D.) | 233.37 ± 149.81 | 220.27 ± 151.60 | 0.745^(b)^ |
| ALT (Mean ± S.D.) | 161.31 ± 94.29 | 167.87 ± 154.97 | 0.862^(b)^ |
| ALP (Mean ± S.D.) | 659.52 ± 462.14 | 623.03 ± 404.85 | 0.744^(b)^ |
| **Genotype** |  |  |  |
| **rs2501577 (A/G)** |  |  |  |
| AA-AG | 11 (57.89%) | 21 (51.22%) |  |
| GG | 8 (42.11%) | 20 (48.78%) | 0.629 ^(a)^ |
| **rs11194981 (C/T)** |  |  |  |
| CC-CT | 13 (68.42%) | 24 (58.53%) |  |
| TT | 6 (31.58%) | 17 (41.47%) | 0.463^(a)^ |
| **rs6761893 (T/A)** |  |  |  |
| TT-TA | 16 (84.21%) | 36 (87.80%) |  |
| AA | 3 (15.79%) | 5 (12.2%) | 0.703^(a)^ |
| **rs727878 (T/C)** |  |  |  |
| TT-TC | 13 (68.42%) | 29 (70.73%) |  |
| CC | 6 (31.58%) | 12 (29.27%) | 0.856^(a)^ |

**Abbreviations:** S.D., standard deviation; AST, serum aspartate transaminase; ALT, serum alanine aminotransferase;
ALP, serum alkaline phosphatase
(a)Chi-square test or Fisher exact test; (b)T-test or Wilcoxon signed rank test

**Supplement Table 6** Demographic data of BA cases and healthy controls

|  | **Cases** | **Controls** |
| --- | --- | --- |
| **No. of subjects** | 60 | 179 |
| **Age ± S.D.** | 32 ± 40 days | 58 ± 12 years |
| **Sex** |  | |
| **Male (%)** | 27 (45.0) | 75 (41.9) |
| **Female (%)** | 33 (55.0) | 104 (58.1) |

**Abbreviations:** S.D., standard deviation;

**Supplement Table 7** Multivariate analysis of genotype of rs6761893 and surgical age by Cox-proportional hazard model

|  | **Hazard ratio (95% CI)** | ***P-value*** |
| --- | --- | --- |
| Genotype of rs6761893  (AA vs TT/TA) | 4.25 (1.018-17.7) | 0.047 |
| Age at surgery  (before vs after 60 days) | 0.47 (0.25-0.9) | 0.024 |

**Abbreviations:** CI, confident interval

**
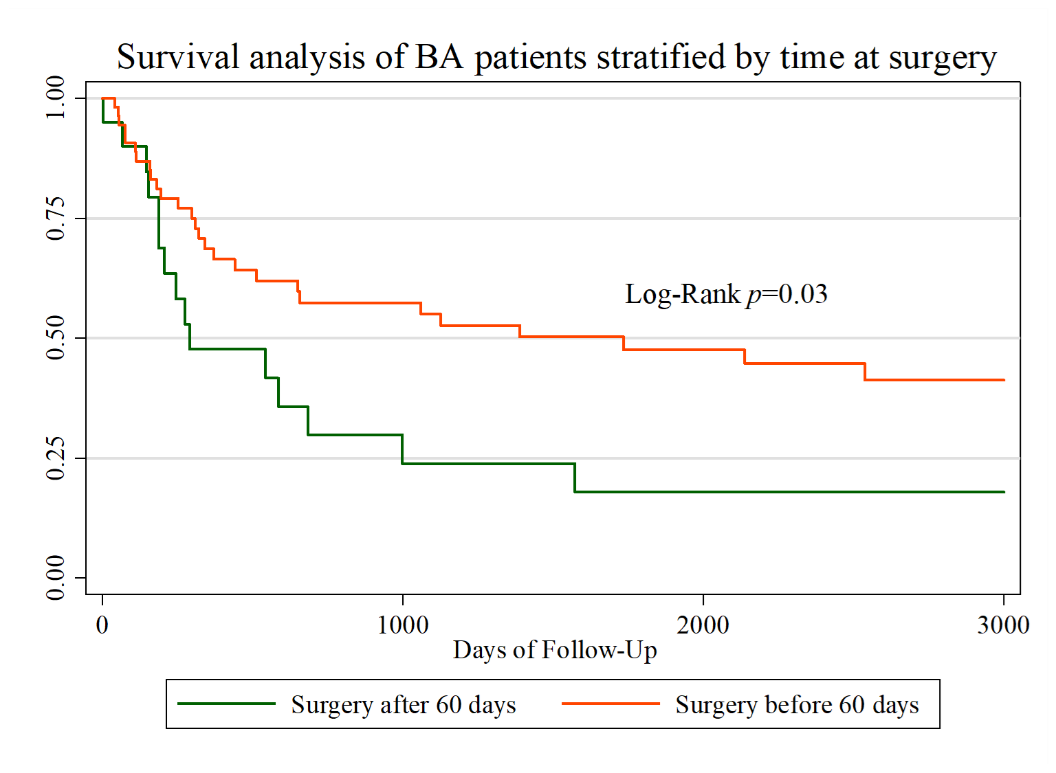
**

**Supplement Figure 1** Kaplan–Meier survival plot of age at Kasai’s operation


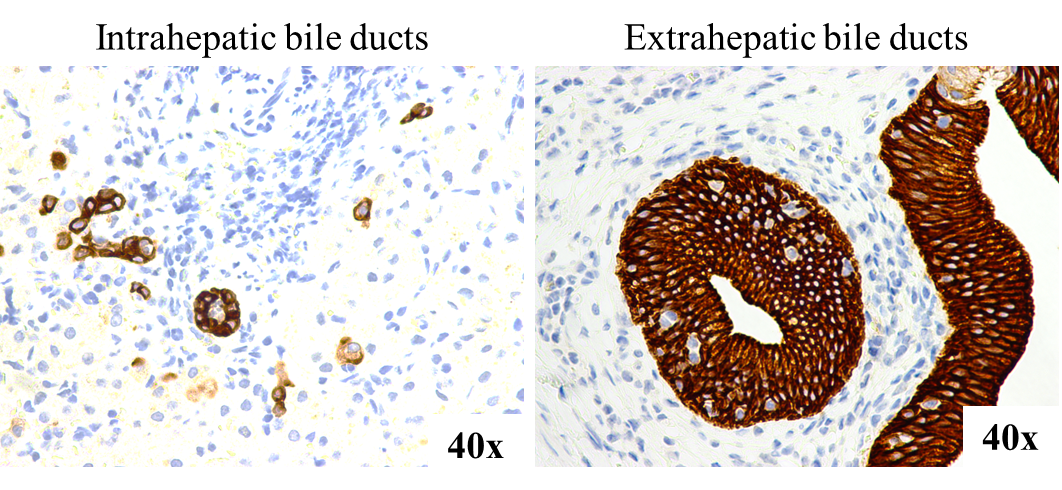


**Supplement Figure 2** Immunohistochemistry staining of CK19 protein in biliary epitheliums for demonstration of their structures
